# Three-Dimensional Assessment of Composite Attachment Wear in Clear Aligner Therapy: A Prospective Cohort Study

**DOI:** 10.7759/cureus.112065

**Published:** 2026-07-04

**Authors:** Zainab Malik, Varun Goyal, Gurkeerat Singh, Raj Kumar Singh, Saba Khan

**Affiliations:** 1 Department of Orthodontics and Dentofacial Orthopaedics, Sudha Rustagi College of Dental Sciences and Research, Faridabad, IND; 2 Department of Orthodontics and Dentofacial Orthopaedics, The Regional Institute of Medical Sciences, Imphal, IND; 3 Department of Orthodontics and Dentofacial Orthopaedics, Dr. Ziauddin Ahmed Dental College and Hospital, Aligarh, IND

**Keywords:** attachment, clear aligners, in vivo, loss, volume

## Abstract

Introduction: Clear aligner therapy relies on composite attachments for precise tooth movement; however, wear may compromise force delivery and treatment efficacy. This study quantitatively assessed morphometric changes in the attachment dimensions and volume over six months and explored patterns according to sex, jaw, and tooth position.

Materials and methods: This prospective cohort study included 12 adults (aged >18 years) with mild-to-moderate malocclusion, contributing 50 attachments bonded with G-ænial Universal Flo (GC Corporation, Tokyo, Japan) composite material. Intraoral scans were obtained post-attachment bonding (T1) and at six months (T6). Attachments were isolated and analyzed using SOLIDWORKS software (Dassault Systèmes SolidWorks Corp., Waltham, Massachusetts) for changes in width, length, height, and volume. Non-parametric tests and linear mixed models were used to evaluate the wear patterns (significance: p < 0.05).

Results: A significant reduction of attachments occurred over six months in all dimensions. The mean width, length, height, and volume decreased from 2.95 (1.90-3.18) to 2.30 (1.40-2.50) mm, 2.25 (2.10-2.50) to 1.60 (1.50-1.90) mm, 1.20 (1.10-1.03) to 1.00 (0.90-1.10) mm, and 9.50 (7.15-10.85) to 7.35 (5.70-8.50) mm³, respectively. The consistent and large Cohen's d effect size indicated a substantial magnitude of change. Posterior attachments showed greater width and volume loss than anterior attachments. Mandibular attachments had higher volume loss than maxillary attachments. No significant sex differences were observed (p > 0.05).

Conclusions: Significant reductions in attachment dimensions and volume were observed over six months, confirming progressive wear during clear aligner therapy. Posterior and mandibular attachments demonstrated greater attrition, highlighting the influence of biomechanical loading and anatomical location. Regular monitoring of attachment integrity and the use of more wear-resistant materials may improve force delivery, treatment efficiency, and clinical predictability.

## Introduction

In orthodontics, clear aligners have emerged as a transformative alternative to traditional fixed appliances, driven by advancements in thermoplastic materials and computer-aided design and manufacturing (CAD-CAM) technology [1]. These devices offer superior esthetics, enhanced patient comfort, and improved oral hygiene, making them increasingly popular for the treatment of various malocclusions [2]. Unlike conventional braces, clear aligners provide discreet treatment, minimizing social stigma and aligning with the growing demand for minimally invasive orthodontic solutions [1,2]. However, their efficacy relies heavily on auxiliary components, particularly composite resin attachments, which enhance force delivery and aligner retention and facilitate precise tooth movements, such as rotation, extrusion, and intrusion [3].

Attachments, typically bonded to tooth surfaces using flowable composites, act as mechanical anchors that effectively transmit orthodontic force. While maintaining structural integrity throughout treatment, they face dual challenges: preserving aesthetics and resisting wear [4]. Esthetically, attachments should exhibit high translucency, shade matching, and stain resistance to beverages such as coffee or wine, ensuring invisibility. Durability is equally critical because attrition can compromise forced delivery, leading to suboptimal outcomes or prolonged treatment. Factors such as occlusal forces, frequent aligner removal, and oral habits exacerbate wear, potentially altering the attachment geometry and reducing treatment predictability [4,5].

Historical research on attachment wear has predominantly involved in vitro simulations and qualitative in vivo assessments. For instance, Barreda et al. [6] utilized scanning electron microscopy to observe surface texture changes in attachments over six months and noted the superior wear resistance of the Filtek Z350 XT composite (3M, Eagan, Minnesota). Lin et al. [7] reported 14.79% for the FC (Filtek Z350XT Flowable Restorative; 3M, Eagan, Minnesota) and 9.70% for the PC (Filtek Z350XT Universal Restorative; 3M, Eagan, Minnesota) via visual inspection in the first year, while Chen et al.'s [8] in vitro study highlighted greater volume loss in flowable composites. More recently, Fausto da Veiga Jardim et al. [5] and Li and Yang [4] employed 3D superimposition to quantify wear; however, neither did they focus on bond failure or surface wear without detailed three-dimensional (breadth, length, and height) or volumetric analysis over time, nor did they thoroughly explore risk factors, such as sex, jaw type (maxillary vs. mandibular), or attachment site (anterior vs. posterior).

Prior research on clear aligners has largely been limited to qualitative in vivo assessments or in vitro simulations, such as Barreda et al.'s [6] scanning electron microscopy analysis or Chen et al.'s [8] wear resistance tests, which reported lower wear volumes; however, these studies lacked real-world applicability and clinical relevance. This prospective clinical study addressed these limitations by evaluating the wear of attachments in patients undergoing clear aligner therapy. The primary aim of this study was to quantitatively assess changes in attachment dimensions (width, length, and height) and volume from post-attachment bonding (T1) till six months (T6) using intraoral scanning and 3D modeling. The secondary objectives included subgroup analyses based on sex, jaw position (maxillary vs. mandibular), and tooth position (anterior vs. posterior) to identify the risk factors for wear, thereby informing strategies to enhance aligner efficacy and longevity.

## Materials and methods

Study design and setting

This prospective observational cohort study was conducted in accordance with the Strengthening the Reporting of Observational Studies in Epidemiology (STROBE) guidelines [9]. The study was conducted in the Department of Orthodontics and Dentofacial Orthopaedics, Sudha Rustagi College of Dental Sciences and Research, Faridabad, India. Participants were recruited consecutively from the outpatient orthodontic clinic between March 2024 and March 2025, with follow-up assessments extending up to six months post-treatment. All procedures were performed in a controlled clinical environment using standard orthodontic protocols to ensure consistency in treatment delivery and data collection.

The study protocol was approved by the Institutional Ethics Committee prior to commencement (IEC/2023/02/15 dated 11/07/2023). All participants provided written informed consent after being informed of the study objectives, procedures, potential risks, benefits, and their right to withdraw at any time without prejudice to their treatment. This study adhered to the principles outlined in the Declaration of Helsinki (2013 revision) and complied with local regulatory requirements for human research [10].

Participants and eligibility criteria

Eligible participants were adults aged >18 years seeking clear aligner therapy for mild-to-moderate malocclusions, defined as crowding or spacing of up to 6 mm based on Little's Irregularity Index [11] or arch length discrepancy measurements. The inclusion criteria were as follows: mild-to-moderate crowding or spacing requiring rectangular attachments placed vertically or horizontally; good oral hygiene, as assessed by an Oral Hygiene Index-Simplified (OHI-S) score < 1.2 [12]; commitment to regular follow-up appointments; overbite of 2-3 mm; and underlying Class I dentoskeletal pattern (ANB of 0-4^0^) with Angle’s Class I molar relationship. The exclusion criteria were as follows: mixed dentition; presence of crown restorations or prosthetic replacements; active periodontal disease (probing depth >4 mm or clinical attachment loss >2 mm); and self-reported or clinically diagnosed bruxism confirmed via occlusal wear facets or patient history.

Participants were sourced from routine clinic referrals, with no incentives provided. A total of 12 patients (50 attachments) were enrolled after screening 18 potential candidates (six were excluded due to bruxism or poor hygiene). Each attachment was treated as an independent unit of analysis to account for intraindividual variability, as multiple attachments per patient were included. All attachments were bonded by a single experienced orthodontist (with >10 years of practice) using standardized techniques to minimize operator bias.

Variables and outcomes

The primary outcome was the change in attachment dimensions (breadth (x-axis), length (y-axis), and height (z-axis)) and volume over time, measured at two time points: T1 (immediately post-attachment bonding) and T6 (six months post-treatment initiation). The secondary outcomes included subgroup analyses of wear patterns based on the following predictors: sex (male/female), jaw type (maxillary/mandibular), and attachment site (anterior [incisors/canines] vs. posterior [premolars/molars]). Potential confounders, such as occlusal forces or dietary habits, were not directly measured but were mitigated through the inclusion/exclusion criteria and patient instructions.

Data sources and measurement

Clear aligner therapy was administered using aligners fabricated from Duran thermoplastic sheets (Scheu Dental GmbH, Iserlohn, Germany) thermoformed over 3D-printed models. Attachments were bonded using G-ænial Universal Flo flowable composite (GC Corporation, Tokyo, Japan) applied via aligner templates for precise placement. Patients were instructed to wear the aligners for 21-22 hours daily, change them biweekly, and remove them during meals, followed by immediate brushing to maintain hygiene. All 50 attachments remained intact, with no losses recorded during the study period, ensuring complete data collection.

Intraoral scans were acquired using a Medit i700 intraoral scanner (Medit Corp., Seoul, South Korea) with an accuracy of up to 11 μm, according to the manufacturer specifications and validated literature [13]. Scans were performed by a calibrated operator under standardized lighting and positioning to ensure reproducibility. The digital models were exported in the stereolithography (STL) format.

**Figure 1:**
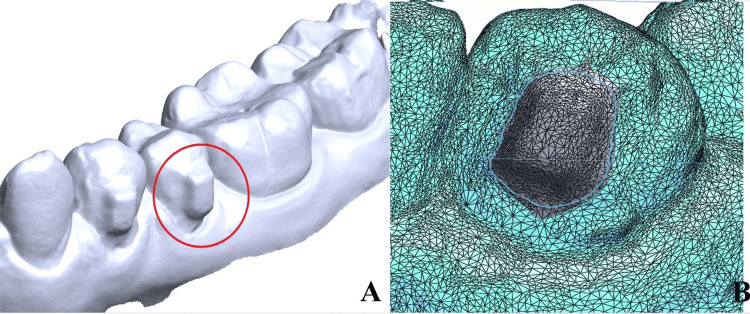
Workflow for three-dimensional attachment isolation and volumetric assessment using SOLIDWORKS software (Dassault Systèmes SolidWorks Corp., Waltham, Massachusetts). (A) Imported stereolithography (STL) model obtained from intraoral scanning (attachment seen in red circle). (B) High-resolution mesh generation and refinement to ensure geometric fidelity. Original images from the study.

For data processing, STL files were imported into SOLIDWORKS software (Dassault Systèmes SolidWorks Corp., Waltham, Massachusetts) to convert them into CAD models. Each attachment was isolated from the surrounding tooth surface using surface trimming and boundary refinement tools to generate a closed, high-resolution three-dimensional model (Figure 1A).

**Figure 2:**
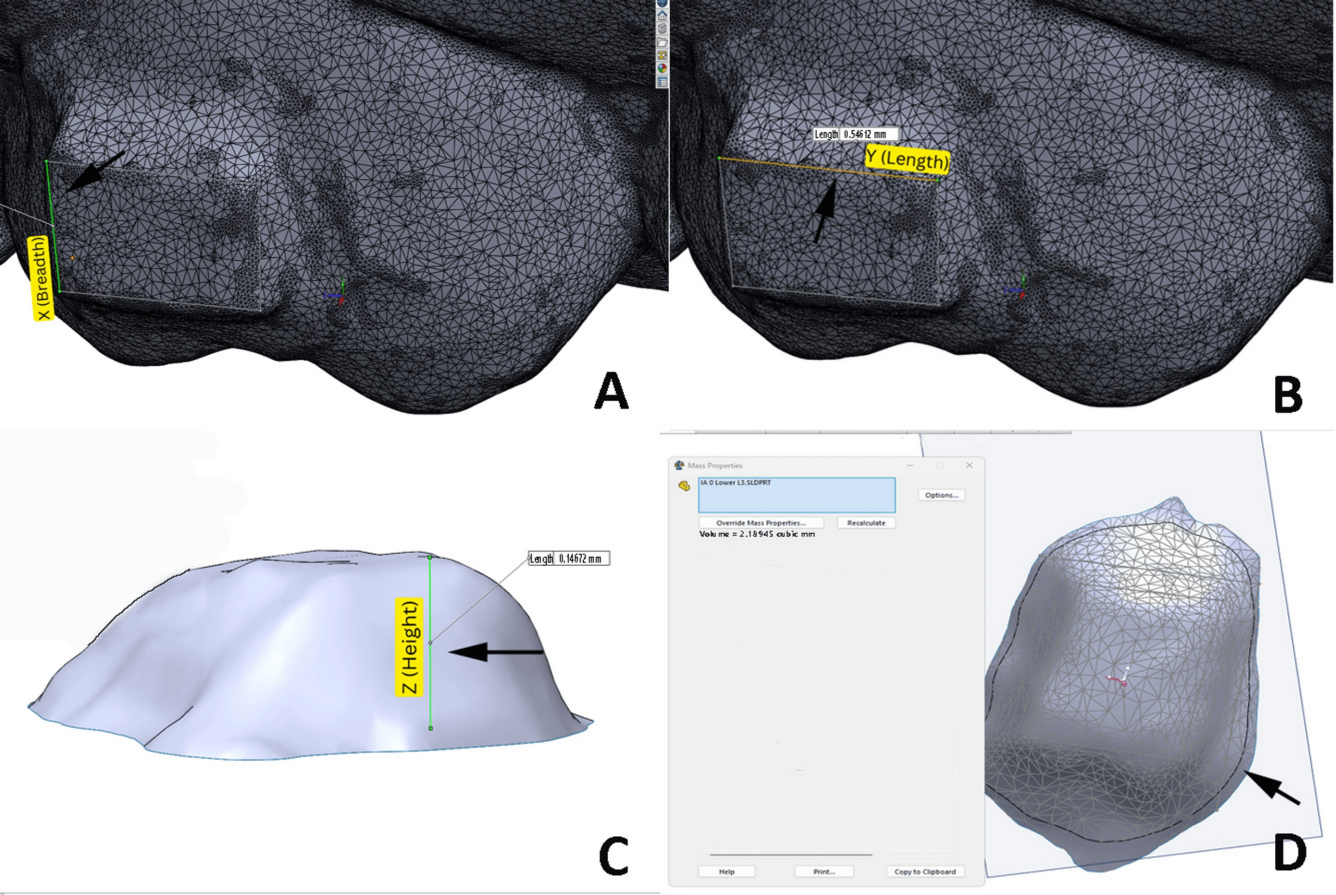
Three-dimensional assessment of linear attachment dimensions using SOLIDWORKS software (Dassault Systèmes SolidWorks Corp., Waltham, Massachusetts). (A) Measurement of attachment width along the x-axis, defined as the maximum cervico-incisal/occluso-gingival dimension. (B) Measurement of attachment length along the y-axis, defined as the maximum mesiodistal dimension. (C) Measurement of attachment height along the z-axis, defined as the maximum buccolingual dimension measured perpendicular to the attachment base. (D) Volume calculation using mass properties. Original images from the study.

Mesh generation and refinement were performed to ensure geometric fidelity while minimizing the surface artifacts (Figure 1B). The same trimming workflow and tolerance parameters were applied across all time points to ensure that the observed differences reflected true material loss rather than segmentation errors. The occlusal (top) surface of each attachment was analyzed relative to a refined mesh. Linear morphometric measurements were performed along a standardized local coordinate system aligned with the attachment geometry (Figure 2A-C).

The following parameters were recorded: width (x-axis) as the maximum cervico-incisal/occluso-gingival dimension of the attachment (Figure 2A); length (y-axis) as the maximum mesiodistal dimension of the attachment (Figure 2B); and height (z-axis) as the maximum buccolingual dimension measured perpendicular to the attachment base (Figure 2C). Measurements were obtained by marking the reference points on the refined attachment surface using the SOLIDWORKS (Dassault Systèmes SolidWorks Corp., Waltham, Massachusetts) measurement tool. Each measurement was repeated twice, and the average was recorded to reduce intra-observer variability. Tools, including 2D/3D sketches, surface modeling, and evaluators, were utilized, with measurements in millimeters (mm) to eight decimal places for precision.

Prior to attachment isolation, serial STL models from each time point were aligned using a surface best-fit registration based on the stable crown surfaces of the corresponding tooth to standardize spatial orientation between scans. Following registration, a consistent boundary plane was established at the tooth-attachment interface, and the attachment was isolated using the surface trim, intersect, and boundary refinement tools in SOLIDWORKS (Dassault Systèmes SolidWorks Corp., Waltham, Massachusetts). The same segmentation protocol was applied to all time points to minimize operator-dependent variability and ensure that measured volumetric changes represented true attachment wear rather than positional changes associated with orthodontic tooth movement.

Volumetric analysis was performed by isolating each attachment as a closed solid body and computing its volume using the mass properties function in SOLIDWORKS (Dassault Systèmes SolidWorks Corp., Waltham, Massachusetts, Figure 2D). Volumes were recorded in cubic millimeters (mm³). Care was taken to maintain consistent attachment boundaries across all time points to ensure accurate and longitudinal comparisons. All measurements were performed by a single blinded assessor (who was unaware of the scan dates and patient details) to prevent detection bias. Intraobserver reliability was assessed using intraclass correlation coefficients (ICC >0.90) for all dimensions.

Addressing bias

Consecutive sampling was used to minimize selection bias. Performance bias was reduced by standardizing attachment placement and patient instructions. Detection bias was assessed by blinding the operator. Attrition bias was low, with no participant loss during follow-up. Reporting bias was mitigated by prespecifying all outcomes in the protocol.

Sample size

The sample size was calculated using G*Power software version 3.1.9.7 (Heinrich Heine University Düsseldorf, Düsseldorf, Germany). Based on an effect size of 0.35, derived from a previous study by Li et al. [4] that compared composite attachment volume across multiple time points (T0: 6.20 ± 3.47 mm^3^; T5: 5.28 ± 3.22 mm^3^), a minimum of 50 composite attachments was required to achieve 80% statistical power with a 5% alpha error.

Statistical analysis

Data were analyzed using IBM SPSS Statistics for Windows, Version 26 (Released 2019; IBM Corp., Armonk, New York). Descriptive statistics were expressed as mean ± standard deviation (SD) and 95% confidence intervals (CI) for changes in the width, length, height, and volume over time. The normality of the data was assessed using the Shapiro-Wilk test. As the distribution of several variables deviated from normality, non-parametric methods were applied for the unadjusted comparisons. Absolute changes from T1 to T6 were assessed using the related-samples Wilcoxon signed-rank test. Exploratory, unadjusted comparisons of six-month changes between demographic and anatomical subgroups (sex, jaw, and attachment site) were performed using the Mann-Whitney U test and were interpreted descriptively. Linear mixed model (LMM) analysis was employed as the primary inferential method to evaluate the effects of time, attachment site (anterior/posterior), jaw (maxillary/mandibular), and sex on morphometric change. Random effects were specified to account for the clustering of multiple attachments within patients and repeated measurements over time. Model assumptions were assessed at the level of the residuals rather than the raw data, and inspection confirmed the approximate normality of the residuals. LMMs were selected because they appropriately handle the hierarchical structure of the data and account for the non-independence of observations, thereby reducing the risk of Type I error inflation. All statistical tests were two-tailed, and a p < 0.05 was considered statistically significant. No missing data were observed, and no sensitivity analyses were required because of complete follow-up.

## Results

The study cohort comprised 50 attachment points from 12 patients with a mean age of 24.25 ± 4.61 years and a female predominance. Attachments at the posterior sites were more common than those at the anterior sites. The distribution of attachments between the maxilla and the mandible was nearly equal (Table 1).

**Table 1:** Demographic and site-wise distribution of attachments included in the study. Age is presented as mean ± standard deviation (SD); other variables (sex, site, and jaw) are expressed as frequency and percentage (n, %).

Variable		Value
Age, mean ± SD	Years	24.25 ± 4.61
Sex, n (%)	Male	5 (41.7%)
	Female	7 (58.3%)
Attachment site, n (%)	Anterior	16 (32.0%)
	Posterior	34 (68.0%)
Attachment jaw, n (%)	Maxilla	26 (52.0%)
	Mandible	24 (48.0%)

Table 2 outlines the average morphometric changes in dental attachment dimensions (width, length, height, and volume) over six months, grouped by sex, jaw, and site. The greatest decrease in width was observed in the posterior attachments and in males. Length changes were similar, ranging from 0.60 mm (females) to 0.50 mm (males). Height reductions were smaller in posterior teeth and in the mandibular arch. Volume decreased the most in the mandible, in the posterior teeth, and in males. This suggests slightly greater wear in the posterior sites and mandible, which is likely due to the biomechanical differences.

**Table 2:** Mean morphometric changes in attachment dimensions after six months according to sex, jaw, and site. Data represent the mean ± SD of changes from post-attachment bonding (T1) to six months (T6). SD: standard deviation.

Time points	Male	Female	Maxilla	Mandible	Anterior	Posterior
	Median (IQR)	Median (IQR)	Median (IQR)	Median (IQR)	Median (IQR)	Median (IQR)
T1-T6 width (mm)	0.50 (0.40-0.68)	0.70 (0.40-0.70)	0.65 (0.40-0.78)	0.60 (0.48-0.70)	0.40 (0.40-0.43)	0.70 (0.60-0.70)
T1-T6 length (mm)	0.50 (0.50-0.60)	0.60 (0.50-0.63)	0.60 (0.50-0.60)	0.55 (0.50-0.63)	0.60 (0.50-0.60)	0.60 (0.50-0.60)
T1-T6 height (mm)	0.20 (0.13-0.30)	0.20 (0.10-0.20)	0.20 (0.20-0.30)	0.10 (0.10-0.20)	0.25 (0.10-0.30)	0.20 (0.10-0.20)
T1-T6 volume (mm^3^)	2.10 (1.40-3.08)	1.95 (1.20-2.65)	1.50 (1.20-2.23)	2.60 (1.60-3.60)	1.75 (0.90-3.30)	2.20 (1.33-2.60)

The pattern of total wear differs significantly across anatomical and demographic subgroups. For location, posterior teeth exhibited significantly greater width loss compared to other parameters (p = 0.001). By arch, maxillary teeth demonstrated significantly greater height reduction (p = 0.003) and mandibular teeth showed greater volume loss (p = 0.002) compared to other dimensions. Regarding sex, neither males nor females showed statistically significant differences in wear patterns across any parameters (Table 3).

**Table 3:** Comparison of overall average wear (T1-T6) of attachment between subgroups for parameters with Wilcoxon signed-rank test. *p < 0.05 denotes statistical significance.

Parameters	Width		Length		Height		Volume	
	W stats	p-value	W stats	p-value	W stats	p-value	W stats	p-value
Male	233.5	0.267	203.0	0.076	217.0	0.132	265.0	0.641
Female								
Anterior	81.5	0.001*	269.0	0.949	189.0	0.076	245.5	0.581
Posterior								
Maxilla	271.0	0.416	299.0	0.794	166.5	0.003*	155.0	0.002*
Mandible								

The Wilcoxon signed-rank test revealed a statistically significant reduction in all morphometric parameters over the six-month period (p = 0.001 for all variables). The mean width, length, height, and volume decreased from 2.95 (1.90-3.18) to 2.30 (1.40-2.50) mm, 2.25 (2.10-2.50) to 1.60 (1.50-1.90) mm, 1.20 (1.10-1.03) to 1.00 (0.90-1.10) mm, and 9.50 (7.15-10.85) to 7.35 (5.70-8.50) mm³, respectively. The consistent and large Cohen's d effect size indicated a substantial magnitude of change. These results indicate that the attachments underwent significant morphometric attrition within a relatively short time frame. The uniform and large effect across all dimensions suggests a generalized, non-selective pattern of wear (Table 4).

**Table 4:** Comparison of morphometric parameters of attachments at post-attachment bonding (T1) and six months (T6) using the Wilcoxon signed-rank test. *p < 0.05 denotes statistical significance; Cohen’s d indicates effect size.

Attrition of attachments	T1	T6	W stats	p-value	Cohen's d
	Median (IQR)	Median (IQR)			
Width (mm)	2.95 (1.90-3.18)	2.30 (1.40-2.50)	1275.0	0.001*	0.85
Length (mm)	2.25 (2.10-2.50)	1.60 (1.50-1.90)	1275.0	0.001*	0.87
Height (mm)	1.20 (1.10-1.30)	1.00 (0.90-1.10)	1271.5	0.001*	0.81
Volume (mm^3^)	9.50 (7.15-10.85)	7.35 (5.70-8.50)	1275.0	0.001*	0.82

Based on the LMM analysis presented in Table 5, both time and attachment site emerged as statistically significant predictors across all four morphometric parameters (p < 0.001 for all, except site and height). In contrast, sex and jaw location did not have significant independent effects on any of the measured outcomes (all p > 0.05). These results led to two primary conclusions. First, the highly significant effect of time confirmed a consistent and substantial reduction in the attachment dimensions (width, length, height, and volume) over the study period, indicating a progressive wear. Second, the significant influence of site, particularly the strong effects on width and volume, revealed that posterior attachments experienced significantly greater morphometric attrition than anterior attachments. This suggests that biomechanical forces are not uniformly distributed, with posterior sites subjected to more intense stress, thereby guiding more targeted clinical monitoring and intervention strategies.

**Table 5:** Linear mixed model (LMM) analysis showing the effects of time, site, sex, and jaw on morphometric parameters. *p < 0.05 denotes statistical significance. Time and attachment site were significant predictors of change across parameters.

Analysis of variance (ANOVA) summary	Width		Length		Height		Volume	
Effect	F stats	p-value	F stats	p-value	F stats	p-value	F stats	p-value
Sex (0 = male, 1 = female)	1.55	0.225	4.60	0.059	2.99	0.096	1.19	0.283
Jaw (o = maxilla, 1 = mandible)	1.55	0.246	3.24	0.106	3.63	0.100	0.32	0.585
Site (0 = anterior, 1 = posterior)	41.19	< 0.001*	12.24	0.005*	5.09	0.050	19.72	0.001*
Time	49.96	< 0.001*	96.99	< 0.001*	31.74	< 0.001*	29.50	< 0.001*

## Discussion

The progressive morphometric attrition of composite attachments in clear aligner therapy highlights the complex interplay between material properties, biomechanical forces, and intraoral environmental factors. The dimensional and volumetric losses observed align with the inherent limitations of flowable composites, which, despite their ease of application and adaptability to tooth surfaces, possess lower filler content and thus reduced mechanical strength than universal restoratives [14]. The G-ænial Universal Flo (GC Corporation, Tokyo, Japan) composite is a low-viscosity, flowable nanohybrid composite resin containing strontium glass and prepolymerized fillers with an average filler size of ~200 nm and a filler loading of approximately 69 wt% (53 vol%). Ocak et al. [15] did an in vitro study to compare the wear resistance of aligner horizontal-rectangular attachments bonded using four different composites (Flow Tain, Transbond XT, G-aenial Universal Flo, and Filtek Z350 XT; GC Corporation, Tokyo, Japan) and found that G-aenial Universal Flo (GC Corporation, Tokyo, Japan) showed the lowest wear resistance (0.35 ± 0.35 mm^3^ of attachment volume difference). Li and Yang [4] reported a 0.815 mm^3^ attachment volume change after eight months, and Chen et al. [8] reported 0.75 mm^3^ of volume change.

The wear of composite attachments in clear aligner therapy is influenced by multiple factors, including frequent tooth brushing, eating, occlusal forces, aligner removal, and the oral environment, with increased friction from these activities likely exacerbating wear [3-5]. Variations in wear volume across studies may stem from differences in composite resin properties, such as filler size, quantity, geometry, distribution, monomer type, and matrix-filler bonding strength, which can enhance wear resistance when optimized. Discrepancies may arise from variations in scanner precision, superimposition methods, measurement techniques, and software accuracy [16]. Traditional studies using impressions and replicas for scanning may introduce errors, whereas intraoral optical scanning offers a more precise and convenient method for obtaining digital models [16,17].

Cyclic loading from mastication and frequent aligner insertions/removals likely induce surface microcracks, leading to material loss through abrasive wear mechanisms, as described in the tribological models of dental composites [4,8]. The thermoplastic nature of aligners may exacerbate this by generating shear stress at the attachment-aligner interface, thereby promoting fatigue failure over time [5].

The pronounced wear in posterior attachments can be attributed to higher occlusal forces in these regions, where bite forces often exceed 200 N, compared to anterior regions with forces below 100 N [18]. Posterior teeth, which are positioned closer to the temporomandibular joint fulcrum, experience amplified compressive and lateral stresses during function, accelerating attrition [19]. Similarly, the mandibular attachments exhibited greater volumetric changes, likely because of the role of the mandible in absorbing occlusal impacts and its exposure to dynamic movements during speech and swallowing. Similarly, Li and Yang [4] reported higher volume changes in the mandibular posterior attachments.

Sex-based differences were minimal, suggesting that intrinsic factors, such as hormonal influences on saliva composition or enamel hardness, are less influential than extrinsic factors. Similarly, Barreda et al. [6] reported no significant sex disparities in attachment durability, attributing variations primarily to behavioral factors, such as dietary habits, which were controlled for in this study through stringent exclusion criteria. Previous studies have shown that females are more concerned about their oral health than males are [20,21]. Our study reported a greater attachment volume loss in males than in females, though the difference was not statistically significant.

The non-selective wear pattern across the dimensions of the attachments indicates isotropic material degradation, potentially driven by the hydrolytic breakdown of the resin matrix in the aqueous oral environment, consistent with previous studies showing uniform dimensional loss under simulated oral conditions [8,22]. The temporal progression of wear, accelerating between three and six months, suggests a threshold effect, where the initial surface polishing from aligner friction transitions to deeper erosive processes [7]. This trajectory corroborates the longitudinal data from Fausto da Veiga Jardim et al. [5], who noted cumulative volume reductions over extended periods, underscoring the need for material innovations, such as nanoparticle-reinforced composites, to enhance fracture toughness.

The degradation of composite attachments with clear aligners is influenced by a wide array of biological factors. Saliva, a crucial component of the oral environment, plays a pivotal role in the biodegradation of resin-based materials. Saliva is a complex fluid rich in inorganic elements, such as calcium and magnesium, and organic substances, including proteins, enzymes, and glycoproteins. Among the protein components, esterases, which are enzymes that break down ester bonds, are primarily responsible for the breakdown of resin composites, particularly those used in attachments [23]. For instance, human saliva-derived esterase exhibits robust degradation activity against bisphenol A-glycidyl methacrylate (Bis-GMA), a widely used monomer in resin composites and adhesives. Other esterases, such as cholesterol esterase-like and pseudocholinesterase-like activities, have also been identified in saliva, contributing to the breakdown of ester bonds in composite resins and accelerating their degradation over time [24].

Bacterial activity complicates the degradation process. *Streptococcus mutans*, a primary bacterium involved in tooth decay, exacerbates the roughness of Bis-GMA-based resins, particularly when co-cultured with saliva. As *S. mutans* colonizes the resin surface, it produces its own esterase enzymes, which amplify the breakdown of the material, leading to increased shrinkage and a reduction in mechanical properties such as hardness, tensile strength, and elasticity. The roughened surface provides an ideal environment for further bacterial adhesion, creating a vicious cycle of microbial colonization and increased degradation [25]. The biofilm produced by these bacteria accelerates resin breakdown and promotes the production of esterase enzymes, amplifying the degradation of the resin material [26].

In addition to the biological degradation of resin-based materials, there is an emerging concern regarding the leaching of micro- and nanoplastics (MNPs) from these materials into the environment. Studies have shown that MNPs, especially those within the 1-5-micron range, can be released from degraded aligners and resin attachments [27]. One of the key concerns in clear aligner therapy is the repeated replacement of aligners, which typically occurs weekly. This frequent change leads to the continuous renewal of compounds released into the oral cavity. Although the change in aligners helps prevent force relaxation and material wear, it also increases the potential for biologically active substances, such as monomers and degradation products, to leach out with each new aligner [27]. A viable approach to address this issue is the incorporation of shape-memory polymers (SMPs) that do not experience force relaxation over a wider range of movements. SMPs can effectively reduce the need for frequent aligner replacements, as they maintain their structural integrity and force delivery capabilities over a longer period, thus reducing the number of aligners required and minimizing compound release [1]. In addition, introducing fillers, such as graphene particles, into the resin could enhance the aligner material's resistance to wear, reduce attrition, and improve its hardness and modulus, thus mitigating the risks of compound release while extending the lifespan of each aligner [28]. Another solution to this problem is to replace composite resin attachments with antimicrobial and bioactive resin-based polymers that possess hardness comparable to that of aligner material [29]. The risks of indentation, wear, and MNP generation can be significantly reduced by ensuring that the hardness of the attachment material matches that of the aligner. These biomedical polymers can be bonded directly to the enamel, eliminating the need for bulk composite material blocks that lead to uneven wear.

The use of 3D intraoral scanning with the Medit i700 intraoral scanner (Seoul, South Korea) provided precise quantification advances beyond qualitative assessments, providing metrics that correlate with clinical predictability. To the best of our knowledge, no previous studies have utilized SOLIDWORKS software (Dassault Systèmes SolidWorks Corp., Waltham, Massachusetts) with its fine mesh analysis method to evaluate 3D attrition and volume wear of orthodontic attachments, as prior research predominantly relies on superimposition techniques (such as Geomagic or MeshLab). This study addresses the gaps in prior research, which often relied on 2D imaging or in vitro simulations, by offering multidimensional in vivo data. Site-specific effects suggest that wear is modulated by load distribution gradients along the dental arch. Integrating these findings with patient-specific factors could refine the predictive algorithms for aligner design and optimize the attachment geometry to mitigate stress concentrations.

Limitations

The small sample size and six-month follow-up period of this study limited insights into long-term wear or applicability to severe malocclusions. The use of a single composite type restricts generalizability, and while confounders such as bruxism were excluded, unmeasured factors such as salivary pH or microbial activity may influence outcomes. The observational design precludes causality and potential measurement errors, despite high intra-observer reliability, and warrants multi-operator validation. Future studies should explore diverse populations and incorporate finite element modeling to elucidate wear dynamics.

## Conclusions

This prospective clinical study provided critical insights into the morphometric attrition of composite attachments in clear aligner therapy, demonstrating significant reductions in width, length, height, and volume over six months. Notably, the posterior and mandibular attachments exhibited the most pronounced wear, as quantified by precise intraoral scanning and 3D modeling. These results underscore the significant influence of anatomical positioning and biomechanical forces, with the posterior and mandibular regions experiencing greater occlusal stresses, driving accelerated attrition. These findings advocate targeted clinical strategies, such as adopting reinforced composite materials or optimized attachment designs, to enhance aligner efficacy and treatment precision. Despite limitations, including a small sample size and six-month follow-up, this study emphasizes the need for advanced material innovations and regular monitoring to improve the durability and effectiveness of clear aligner therapy, ultimately advancing patient-centered orthodontic outcomes.
